# Broadband high-resolution X-ray ptychography system spanning tender to hard X-ray regimes

**DOI:** 10.1107/S2052252525009236

**Published:** 2026-01-01

**Authors:** Yuhei Sasaki, Nozomu Ishiguro, Masaki Abe, Shuntaro Takazawa, Hideshi Uematsu, Naru Okawa, Fusae Kaneko, Yukio Takahashi

**Affiliations:** a International Center for Synchrotron Radiation Innovation Smart (SRIS) Tohoku University Sendai980-8572 Japan; b Department of Metallurgy, Graduate School of Engineering Tohoku University Sendai980-8579 Japan; c RIKEN SPring-8 Center, Hyogo679-5148, Japan; d Institute of Multidisciplinary Research for Advanced Materials (IMRAM) Tohoku University Sendai980-8577 Japan; e Sumitomo Rubber Industries Ltd Kobe Hyogo651-0071 Japan; f Institute for Materials Research Tohoku University Sendai980-8577 Japan; UCL, United Kingdom

**Keywords:** tender/hard X-ray ptychography, advanced Kirkpatrick–Baez focusing mirrors, CITIUS detectors, X-ray spectromicroscopy, NanoTerasu

## Abstract

X-ray ptychography is a high-resolution imaging technique capable of resolving nanoscale structures and probing chemical states. However, despite advances, most conventional X-ray ptychography systems have been designed to operate within a single energy regime, such as hard (> 5 keV), soft (< 2 keV) or tender (2–5 keV) X-rays. We have developed a high-efficiency achromatic ptychographic measurement system on beamline BL10U at NanoTerasu, a 3 GeV synchrotron radiation facility in Japan, that incorporates advanced Kirkpatrick–Baez focusing mirrors and a high-speed CITIUS detector. Utilizing this innovative system, we have successfully reconstructed phase images with a spatial resolution exceeding 50 nm from 200 nm-thick tantalum test charts at X-ray energies of 2.5, 5.0 and 7.5 keV. We have performed energy scans around the Ca and S *K* edges on CaSO_4_·2H_2_O particles to demonstrate the spectromicroscopic capabilities of the system. The reconstructed images provide spatially resolved X-ray absorption spectra, revealing distinct edge features. This system not only enables element-specific imaging across the tender to hard X-ray spectrum but is also particularly advantageous for light-element materials, which benefit from enhanced phase contrast in the tender X-ray range. This advancement opens up new possibilities for *in situ* imaging of complex chemical environments in energy devices and biological specimens.

## Abstract 2

An advanced X-ray ptychography system has been developed that facilitates nanoscale imaging across both tender and hard X-ray regions using a single optical setup.

## 1. Introduction

Tender (2–5 keV) and hard (>5 keV) X-rays possess the necessary penetration power and short wavelengths for non-destructive observation of micrometre-thick samples. This energy range encompasses the *K* absorption edges of 3*p* elements, such as sulfur and phosphorus, as well as 3*d* transition metals like cobalt and nickel. Additionally, it covers the *L* absorption edges of 4*d* transition metals, including tin and rhodium, and lanthanides, such as cerium. These elements are integral to various functional materials, such as nickel in lithium-ion batteries (Bak *et al.*, 2024 ▸), tin in solar cells (Sun *et al.*, 2023 ▸), cerium in catalysts (Hirose *et al.*, 2019 ▸) and sulfur in biological specimens (Deng *et al.*, 2018 ▸), and they have been extensively investigated utilizing tender and hard X-ray microscopy techniques. However, the analysis of these mater­ials necessitates high-spatial-resolution spectromicroscopy that spans a broad X-ray energy range.

Coherent X-ray diffraction imaging (CXDI) is a lensless technique whose resolution is not constrained by optical elements (Chapman & Nugent, 2010 ▸; Miao, 2025 ▸). Among CXDI techniques, X-ray ptychography stands out as a scanning approach that reconstructs both the sample image and the probe wavefront from far-field diffraction patterns by utilizing phase-retrieval algorithms (Rodenburg *et al.*, 2007 ▸). The maximum scattering angle determines the resolution of this technique. When combined with X-ray absorption fine structure (XAFS), ptychography facilitates energy-resolved chemical-state imaging, a process referred to as spectroscopic ptychography (Giewekemeyer *et al.*, 2011 ▸; Urquhart, 2022 ▸). This spectromicroscopy achieves spatial resolutions of the order of tens of nanometres (Shapiro *et al.*, 2014 ▸; Hitchcock *et al.*, 2024 ▸).

X-ray ptychography was initially developed in the hard X-ray regime (Rodenburg *et al.*, 2007 ▸). Subsequent advancements in the field have incorporated focusing optics, such as Fresnel zone plates (FZPs) and total-reflection mirrors, notably Kirkpatrick–Baez (KB) mirrors (Holler *et al.*, 2014 ▸; Cesar Da Silva *et al.*, 2017 ▸; Deng *et al.*, 2019 ▸; Kahnt *et al.*, 2022 ▸). Soft X-ray ptychography was first investigated around 2011 (Giewekemeyer *et al.*, 2011 ▸); recent implementations have utilized a Wolter mirror (Kimura *et al.*, 2022 ▸). In 2021, tender X-ray ptychography was demonstrated at SPring-8 on beamline BL27SU (Abe *et al.*, 2021 ▸), achieving a resolution of approximately 50 nm at 2.5 keV using an FZP-based system. However, despite these developments, most ptychographic systems have been designed to operate within a single energy range, typically limited to hard, soft or tender X-rays.

In 2024, the operational launch of the 3 GeV synchrotron facility NanoTerasu (Obara *et al.*, 2025 ▸) marked a significant advancement, providing brightness levels that are ten times greater than those of SPring-8 in the tender regime, while also offering comparable brightness in the hard X-ray regime. Tender X-ray ptychography was subsequently demonstrated on the NanoTerasu BL10U beamline, achieving a resolution of approximately 20 nm on a 200 nm-thick tantalum test chart (Ishiguro *et al.*, 2024 ▸). However, the system relies on a chromatic FZP, which necessitates realignment for substantial energy shifts. Moreover, the FZP used in this setup exhibited a low focusing efficiency of only 16% at 3.5 keV, and the utilized detector had a limited quantum efficiency of approximately 33% at the same energy. To harness fully the capabilities of the NanoTerasu light source across the spectrum from tender to hard X-rays, an imaging system must incorporate achromatic high-efficiency total-reflection optics and detectors with high quantum efficiency.

This study proposes the development of an X-ray ptychography system on NanoTerasu BL10U. The system is equipped with high-efficiency achromatic advanced Kirkpatrick–Baez (AKB) mirrors and a CITIUS detector with single-photon sensitivity, wide dynamic range and compatibility across both tender and hard X-rays. We validate the system’s performance through ptychographic measurements conducted on both a test pattern and a real sample.

## 2. Experimental setup

**Figure 1:**
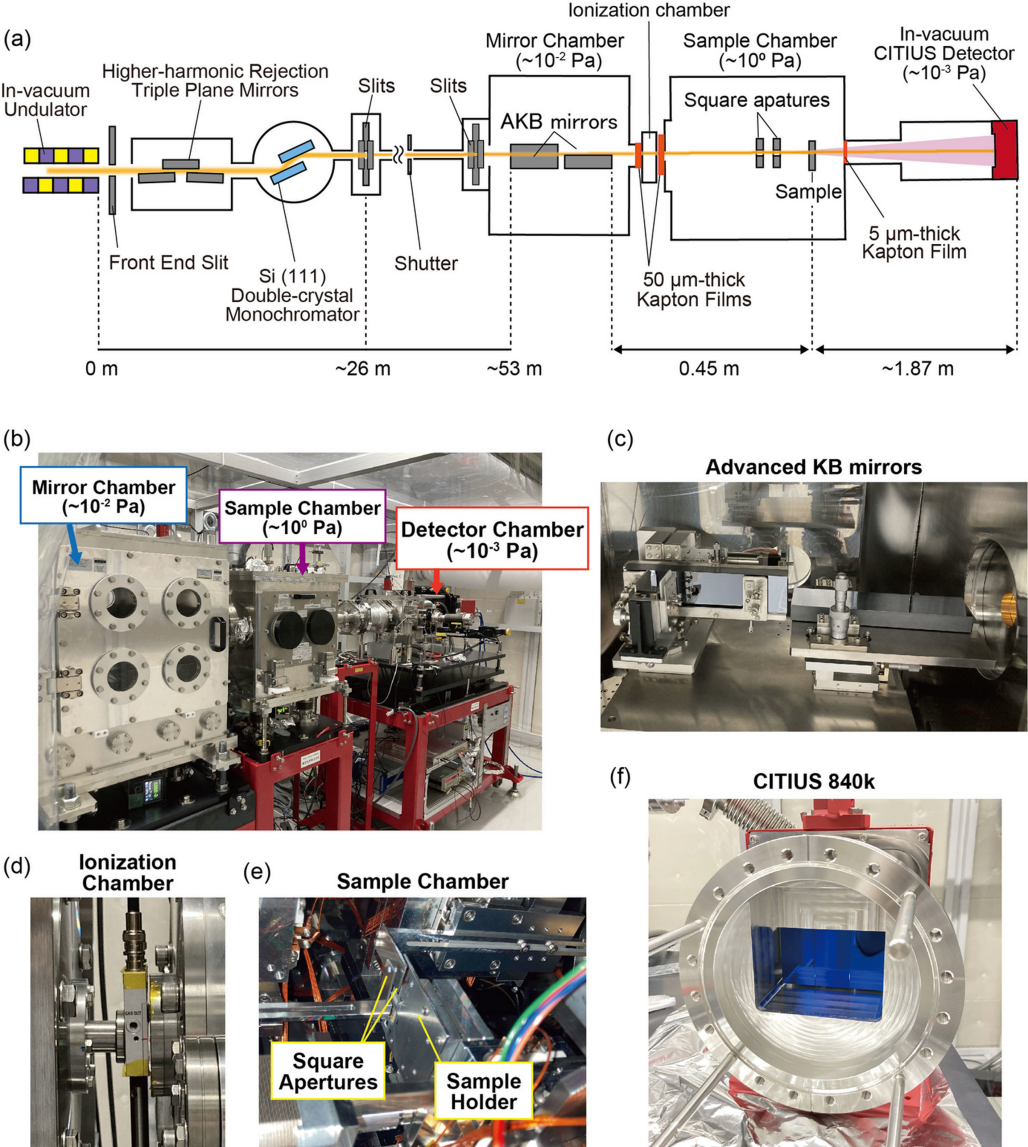
(*a*) Schematic diagram of the optical layout on the NanoTerasu beamline BL10U. (*b*) Photograph of the experimental setup. (*c*) AKB mirrors installed in the vacuum chamber. (*d*) Ionization chamber placed between the mirror and sample chambers for monitoring incident X-ray intensity. (*e*) Interior of the sample chamber, showing the square apertures in front of the sample. (*f*) CITIUS 840k detector utilized for collecting diffraction patterns.

Figs. 1 ▸(*a*) and 1 ▸(*b*) show a schematic diagram and a photograph, respectively, of the experimental setup on BL10U at NanoTerasu. The synchrotron radiation emitted from an in-vacuum undulator was monochromated using higher-harmonic rejection triple-plane mirrors in conjunction with an Si(111) double-crystal monochromator. The first and third mirrors were coated with carbon, whereas the second mirror was coated with nickel. Carbon-coated mirrors efficiently reflect the fundamental radiation in the tender X-ray region while suppressing higher-order harmonics. At 2.5 keV, the grazing-incidence angles were set to 5 mrad for the first and third mirrors, and 10 mrad for the second mirror. For higher photon energies, these angles were adjusted to 4, 8 and 4 mrad, respectively. Beam coherence was maintained by shaping the beam using slits positioned approximately 26 m downstream from the light source.

The AKB mirrors (JTEC Corporation) were situated approximately 53 m downstream from the light source, enabling two-dimensional focusing of the X-rays. These mirrors comprise a pair of one-dimensional focusing mirrors with elliptic and hyperbolic surfaces, commonly referred to as one-dimensional Wolter mirrors (Kodama *et al.*, 1996 ▸; Sauneuf *et al.*, 1997 ▸). They correct for coma aberration and exhibit low sensitivity to variations in the grazing-incidence angle, ensuring stable focusing performance. As the AKB mirrors utilized in this study [Fig. 1 ▸(*c*)] feature a monolithic design that integrates both elliptical and hyperbolic surfaces on a single substrate, the alignment process for focusing is simplified (Matsuyama *et al.*, 2017 ▸). These mirrors share the same design as those developed for the SPring-8 BL29XU beamline (Hirose *et al.*, 2020 ▸). The AKB mirrors demonstrated a reflectivity of approximately 68% at 2.5 keV, significantly higher than the approximately 28% first-order efficiency of the FZP (Abe *et al.*, 2021 ▸). Furthermore, they maintained a reflectivity of approximately 78% within the 5.0–7.5 keV range. These properties make the AKB mirrors a suitable candidate for high-resolution spectromicroscopic imaging (Matsuyama *et al.*, 2019 ▸).

The mirrors were housed within a vacuum chamber, and the upstream slits were adjusted to illuminate only their effective areas. Both the exit of the mirror chamber and the entrance to the sample chamber were sealed with 50 µm-thick Kapton windows. A 10 mm-long ionization chamber (S-2858; Ohyo Koken Kogyo Co. Ltd) was positioned between the two chambers [Fig. 1 ▸(*d*)] and used to monitor the intensity of incident X-rays through the measurement of air ionization current.

The sample was mounted on a piezo stage (P-621.ZLC, P-621.1CL; PI GmbH) and aligned at the focal plane within the in-vacuum sample chamber [Fig. 1 ▸(*e*)]. Two square apertures were installed in front of the sample to suppress parasitic scattering from the focusing optics (Takahashi *et al.*, 2013 ▸). Each aperture was constructed from a 200 µm-thick silicon transmission electron microscopy grid (NT010C; Norcada) from which the SiN membrane had been removed. The sample and detector chambers were connected via a flight tube. A 5 µm-thick Kapton window sealed the sample chamber exit to maintain vacuum conditions (∼10^−3^ Pa) in the detector chamber. Diffraction patterns were collected using a CITIUS detector [Fig. 1 ▸(*f*)] located approximately 1.87 m downstream from the sample. CITIUS exhibits a quantum efficiency of over 97% in the 1.5–5.0 keV range and accommodates photon energies up to 30 keV. This detector can handle approximately 250 Mcounts per second per pixel at 6.5 keV without experiencing saturation (Takahashi *et al.*, 2023 ▸). The timing of the data acquisition was synchronized with both the upstream shutter and the ionization chamber, ensuring precise normalization of intensity measurements. Furthermore, the detector was synchronized with the radio frequency (RF) signal of the accelerator to eliminate beat artefacts caused by timing mismatches. At high frame rates with short exposure times, asynchronous operation between the detector and the RF signal can result in apparent X-ray intensity fluctuations (Nishino *et al.*, 2023 ▸). In this study, the measurements were synchronized and performed with 1 s exposures, thereby avoiding such fluctuations.

## 3. Performance assessment of the measurement system

### 3.1. X-ray ptychography of a 200 nm-thick tantalum test chart

A 200 nm-thick tantalum (Ta) test chart was employed to assess the performance of the measurement system. The storage ring current was 190 mA, approximately half of the planned maximum of 400 mA. The slit dimensions downstream of the monochromator were configured to (horizontal, H) 10 µm × (vertical, V) 50 µm. The photon energies utilized in the experiment were 2.5, 5.0 and 7.5 keV. The photon flux at the sample position, calculated from the ionization chamber current and attenuation caused by the Kapton window, was determined to be 2.5 × 10^7^ photons s^−1^ at 2.5 keV, 1.2 × 10^9^ photons s^−1^ at 5.0 keV and 2.7 × 10^8^ photons s^−1^ at 7.5 keV. Notably, the photon flux exceeded that of FZP optics (Abe *et al.*, 2021 ▸; Ishiguro *et al.*, 2024 ▸). The sample was raster-scanned with a 200 nm step size over a 13 × 13 grid of points overlapping the field of view, with an exposure time of 1 s per scan point. Prior to initiating the first scan point, beam-position drift was corrected using a dark-field knife-edge scan (Suzuki *et al.*, 2005 ▸; Takahashi *et al.*, 2011 ▸). The details of this correction are described in the supporting information, Section S1.

**Figure 2:**
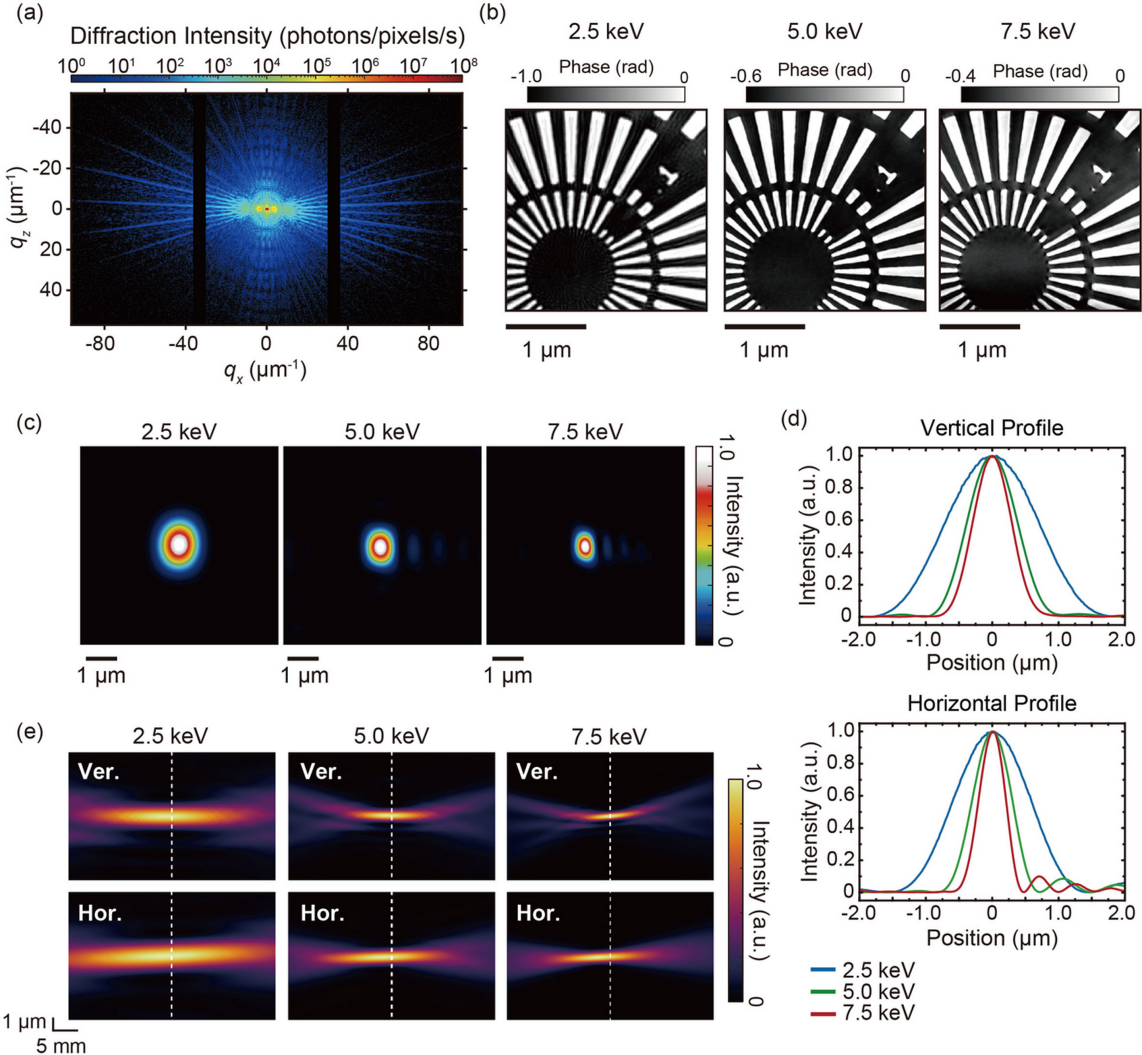
(*a*) Diffraction intensity pattern of the 200 nm-thick Ta test chart at 5.0 keV. (*b*) Reconstructed phase images of the 200 nm-thick Ta test chart at different X-ray energies. (*c*) Intensity distributions of the reconstructed probe functions. (*d*) One-dimensional intensity profiles of the main lobe of the reconstructed probe function, corresponding to the region of highest photon density at the focal spot. (*e*) Beam waists along the vertical (top) and horizontal (bottom) axes. The white dotted lines represent the sample position.

The diffraction intensity pattern of the Ta test chart at 5.0 keV for a scan point is shown in Fig. 2 ▸(*a*). The diffraction pattern comprises 1231 horizontal and 728 vertical pixels, including the sensor gap. An image size of 1231 × 1231 pixels was utilized for the reconstruction to ensure uniform pixel dimensions. Pixels located in the sensor gaps and areas outside the sensor were set to zero. Image reconstruction was performed using the *ePIE* algorithm (Maiden & Rodenburg, 2009 ▸) with two mixed-state modes (Thibault & Menzel, 2013 ▸) incorporating a phase-image-guided filter (Abe *et al.*, 2024 ▸) and probe position correction (Dwivedi *et al.*, 2018 ▸). Seven hundred iterations were conducted, with the phase-guided filter applied during the initial 400 iterations to facilitate convergence. The initial sample image was set to amplitude 1 and phase 0. The initial probe was defined as a sinc function, derived from the rectangular direct beam on the detector. The pixel sizes of the reconstructed images were 10.4 nm at 2.5 keV, 5.19 nm at 5.0 keV and 3.46 nm at 7.5 keV.

The phase images of the test chart at 2.5–7.5 keV are shown in Fig. 2 ▸(*b*). All images indicated the successful resolution of even the smallest structure of 50 nm. The spatial resolution of the phase images was assessed using the phase retrieval transfer function (PRTF) (Chapman *et al.*, 2006 ▸). The full-period spatial resolution was defined as the inverse of the spatial frequency at which the PRTF curve intersected the 1/e threshold. The spatial resolutions were 38.7 nm at 2.5 keV, 13.4 nm at 5.0 keV and 16.1 nm at 7.5 keV. Details of the resolution evaluations based on the PRTF and edge-profile analyses are provided in the supporting information, Sections S3 and S4, respectively, and show good agreement.

These findings underscore the system’s practical applicability as a nanoscale imaging technique. Fig. 2 ▸(*c*) presents the intensity distributions of the reconstructed probe at 2.5–7.5 keV, obtained by summing the two probe modes. The first mode accounted for over 92% of the total photon count in all instances. The amplitude and phase images of the first mode are shown in Figs. S3(*a*) and S3(*b*), respectively, in the supporting information. At all energy points, a main beam and several side lobes were observed. One-dimensional intensity profiles of the main beam are shown in Fig. 2 ▸(*d*). The spot sizes were assessed using the full width at half-maximum, yielding measurements of (H)1.26 µm × (V)1.54 µm at 2.5 keV, (H)0.66 µm × (V)0.84 µm at 5.0 keV and (H)0.44 µm × (V)0.66 µm at 7.5 keV. These spot sizes closely align with the focused beam sizes predicted by the mirror design parameters.

To validate the achromaticity of the AKB mirrors, beam waists at 2.5–7.5 keV were obtained by numerically propagating the probe function using the angular spectrum method. The beam waists at each energy are shown in Fig. 2 ▸(*e*). The focus position remained stable near the sample across all three energy points, from 2.5 to 7.5 keV. When the X-ray energy significantly changes, FZP-based systems (Ishiguro *et al.*, 2024 ▸) require realignment of the focal position. However, owing to the achromaticity of the AKB mirrors, the system can maintain its focus throughout energy scans following a single mirror alignment. These results underscore the system’s capability for high-resolution broadband nanoscale imaging with stable focusing across a wide energy range.

### 3.2. Tender X-ray spectroscopic ptychography of CaSO_4_·2H_2_O particles

X-ray spectroscopic ptychography measurements were conducted on CaSO_4_·2H_2_O particles near the Ca *K* edge and S *K* edge. The CaSO_4_·2H_2_O powder in this study was a commercial product of Nacalai Tesque Inc. This compound has a monoclinic crystal structure with a *C*2/*c* space group (Boeyens & Ichharam, 2002 ▸). The powder was dispersed in aceto­nitrile, deposited onto an SiN membrane window (Norcada, NX7150E) and air-dried. Fig. S5(*a*) shows a scanning electron microscopy (SEM) image of the sample immediately after deposition onto the membrane.

Ptychography measurements were repeatedly performed while sweeping the photon energy. The post-monochromator slit dimensions were set to (H)10 µm × (V)30 µm to ensure probe stability. The sample underwent raster scanning across a 19 × 19 grid with a spacing of 200 nm, with an exposure time of 1 s per scan point. Prior to each energy measurement, beam-position shifts were corrected using a dark-field knife-edge scan.

Twenty-four and twenty-two X-ray energy points were measured near the Ca *K* edge (4.000–4.0932 keV) and S *K* edge (2.226–2.500 keV), respectively. During the energy scans, the photon flux at the sample position was 1.8 × 10^8^ photons s^−1^ near the Ca *K* edge and 1.3 × 10^7^ photons s^−1^ near the S *K* edge. Phase retrieval was conducted using the *ePIE* algorithm, incorporating position corrections and the Kramers–Kronig relation constraint (Hirose *et al.*, 2017 ▸). The initial sample image was uniformly set to one, whereas the initial probe was set to the probe function reconstructed from the test chart measurement. A total of 1600 iterations were performed, resulting in reconstructed images with pixel sizes of 6.5 nm and 10.53 nm at the Ca and S *K* edges, respectively.

**Figure 3:**
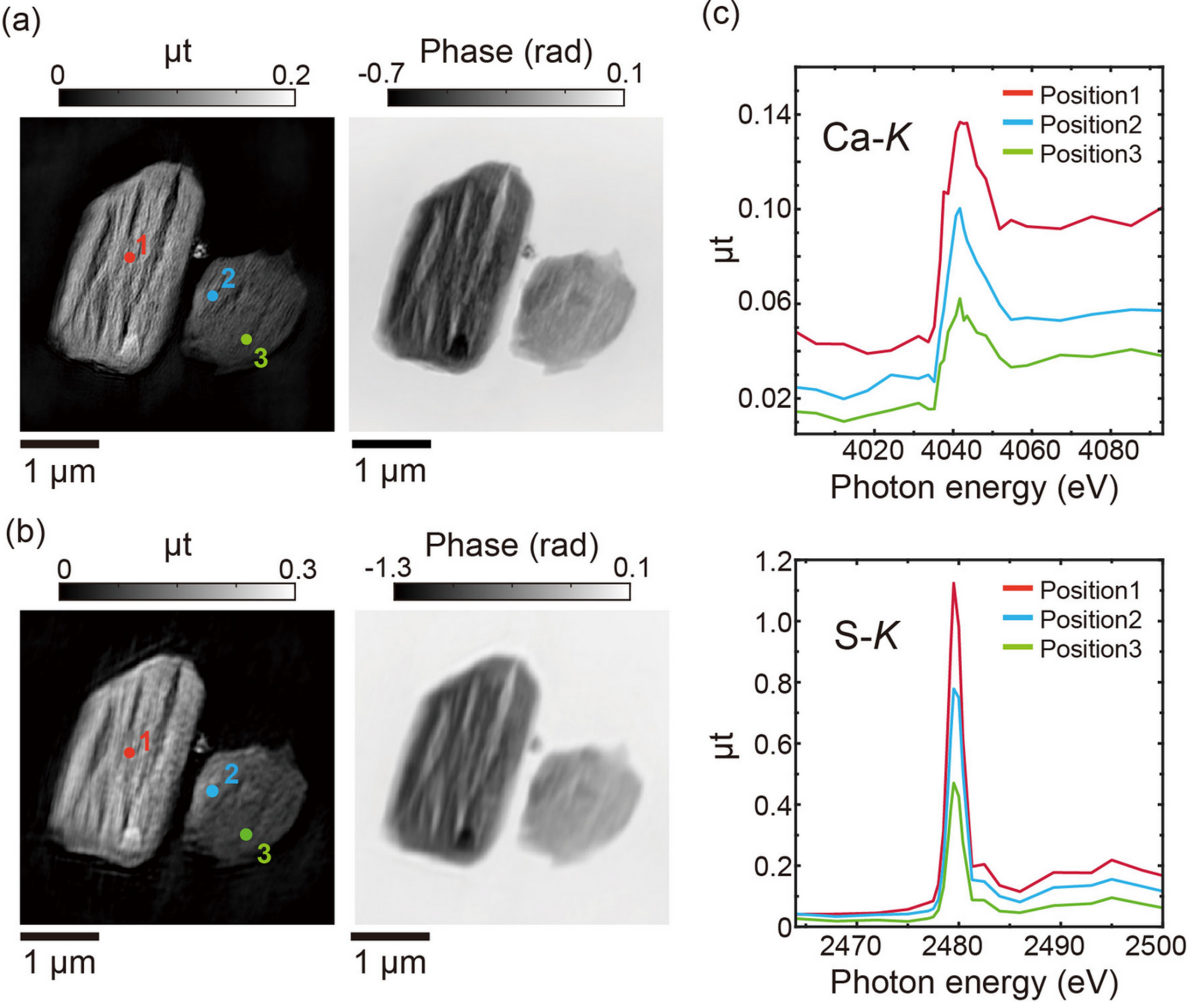
(*a*), (*b*) Reconstructed absorption (left) and phase (right) images of CaSO_4_·2H_2_O particles at 4.0932 keV [(*a*), near the Ca *K* edge] and 2.500 keV [(*b*), near the S *K* edge]. (*c*) Spatially resolved X-ray absorption spectra at the Ca *K* edge (top) and S *K* edge (bottom), extracted from positions 1, 2 and 3 in the absorption images.

The phase and absorption images of CaSO_4_·2H_2_O particles acquired near the Ca and S *K* edges are shown in Figs. 3 ▸(*a*) and 3 ▸(*b*), respectively. The reconstructed images distinctly reveal the particle shapes observed in the SEM image. The spatial resolutions, quantified by the PRTF, were better than 49.3 nm at the Ca *K* edge and 154 nm at the S *K* edge. Fine crack-like structures were observed in the reconstructed images. Fig. S5(*b*) shows a post-measurement SEM image, confirming the presence of these cracks, which were probably formed during the dehydration process (Tang *et al.*, 2019 ▸; Carbone *et al.*, 2008 ▸; Brantut *et al.*, 2012 ▸). Details of the crack formation are provided in Section S5.

X-ray absorption spectra were extracted from the energy stack of absorption images, with extracted regions of 8 × 8 and 15 × 15 pixels at the Ca and S *K* edges, respectively. The spatially resolved spectra extracted at the Ca and S *K* edges are shown in Fig. 3 ▸(*c*). The spectra are similar to those of CaSO_4_·2H_2_O (gypsum) or CaSO_4_ (anhydrite) (Li *et al.*, 1995 ▸; Prietzel *et al.*, 2021 ▸). At the S *K* edge, both gypsum and anhydrite exhibit similar spectral features, and the same trend is observed in the present sample. In contrast, at the Ca *K* edge, differences in the position and width of the white line between gypsum and anhydrite are reported, which are also apparent in Fig. 3 ▸(*c*). Due to limited beam time, the number of energy points and the energy calibration were insufficient for a quantitative analysis of dehydration, which is left for future work. Nevertheless, the present results qualitatively demonstrate the feasibility of the measurements and highlight the capability of the system for nanoscale spectromicroscopy in the tender X-ray regime.

### 3.3. Towards further enhancement of spatial resolution and measurement throughput

Enhancing spatial resolution and measurement throughput is contingent upon increasing the incident X-ray flux. Currently, the storage ring operates at approximately half of its designed maximum current, suggesting the photon flux could potentially double in both hard and tender X-ray regimes in the future. Fully utilizing the source brightness in the hard X-ray regime requires careful consideration of the focusing optics. The reflectivity of the AKB mirrors drops below 10% above 10.3 keV. Thus, an alternative focusing device is recommended above 10 keV. Nonetheless, the AKB mirrors demonstrate reflectivity of the order of 10^−5^ above 12 keV, suppressing higher-order harmonics.

The removal of higher-harmonic mirrors appears feasible above approximately 4 keV, which could significantly increase the flux. At lower energies, the ionization chamber and the window materials reduce the flux. Specifically, at 2.5 keV, a 10 mm air path transmits approximately 72% of the X-rays, whereas two 50 µm-thick Kapton windows transmit only about 5%. Therefore, to enhance the flux, the window mater­ials should be replaced with alternatives offering greater X-ray transparency, while maintaining the use of the ionization chamber. An additional improvement could be realized by substituting the current incident X-ray monitor with one utilizing an SiC membrane (Trovato *et al.*, 2025 ▸). This change would eliminate the need for an air path and Kapton windows.

## 4. Conclusion

By combining AKB mirrors with a CITIUS detector, we have developed a novel ptychographic measurement system on the NanoTerasu BL10U beamline. Owing to the high-efficiency focusing capabilities of total-reflection mirrors and the exceptional quantum efficiency of the CITIUS detector, the photon flux was increased compared with that of conventional tender X-ray ptychography systems that employ FZPs. Ptychography measurements of a 200 nm-thick Ta test chart yielded phase images with spatial resolutions of 38.7 nm at 2.5 keV, 13.4 nm at 5.0 keV and 16.1 nm at 7.5 keV. We also performed tender X-ray spectroscopic ptychography on CaSO_4_·2H_2_O particles near the Ca *K* edge and S *K* edge. The spatially resolved X-ray absorption spectra extracted from the reconstructed images distinctly revealed absorption-edge features at both edges, demonstrating the viability of spectro­microscopy using this system. Enhancing both spatial resolution and measurement throughput could be achieved by considering the removal of higher-harmonics rejection mirrors, and selecting more suitable window materials in the system.

This measurement setup facilitates both tender and hard X-ray ptychography using a single optical configuration. In addition to enabling chemical-state analysis near the *K* and *L* edges, tender X-rays provide high-phase-contrast imaging of samples containing light elements. Exploiting these advantages, the system holds significant potential for visualizing complex chemical states in lithium-ion batteries and catalysts, imaging thick biological cells, and analysing sulfur states in materials such as tyre rubber.

We believe that this technique will serve as a powerful tool for advancing research in materials science, life science and beyond. Its ability to deliver high-contrast imaging of low-*Z*-element materials, combined with its compatibility with *operando* environments, renders it particularly well suited for applications such as *in situ* analysis of energy devices, soft matter and biological specimens.

## 5. Related literature

For further literature related to the supporting information, see Brantut *et al.* (2012 ▸), Carbone *et al.* (2008 ▸) and Tang *et al.* (2019 ▸).

## Supplementary Material

Detailed experimental procedures and additional data. DOI: 10.1107/S2052252525009236/ro5046sup1.pdf
