# Supplementary material for: Broadband high-resolution X-ray ptychography system spanning tender to hard X-ray regimes
Source: IUCrJ. 2026 Jan 1;13(Pt 1):11–8. doi: 10.1107/S2052252525009236 (PMC12809445; doi:10.1107/S2052252525009236)
Supplement: Supplementary file 1 [file m-13-00011-sup1.pdf]

IUCrJ

Volume 13 (2026)

Supporting information for article:

Broadband high-resolution X-ray ptychography system spanning tender to hard X-ray regimes

Yuhei Sasaki, Nozomu Ishiguro, Masaki Abe, Shuntaro Takazawa, Hideshi Uematsu, Naru Okawa, Fusae Kaneko and Yukio Takahashi

S1. X-ray illumination position correction using the dark-field knife-edge scanning method

The position correction using the dark-field knife-edge scanning method is an experimental procedure conducted prior to acquiring the diffraction patterns. A reference point was first determined for each of the horizontal and vertical directions. Examples of reference include a sample edge, a focused ion beam–milled hole or an isolated particle. The reference point was then scanned across the X-ray beam, while diffraction patterns were collected at each position using an image detector. A one-dimensional scattering profile is extracted from each diffraction pattern by isolating the scattering region. This profile is fitted with a Gaussian function to determine the peak position, which corresponds to the reference point location. The measured displacement of the reference point is used to correct the X-ray illumination position by updating the ptychography scan coordinates. This correction is performed independently in the vertical and horizontal directions. By performing this correction periodically, the relative positional deviation between the beam and the sample can be reduced. We refer to this procedure as the origin scan, which enhances the beam positioning accuracy. The origin scan improves the X-ray beam positioning accuracy and maintains a consistent field of view during repeated measurements such as CT and spectroscopic ptychography.

To evaluate the accuracy of this correction, we analysed the field-of-view shift around the first scan position in the reconstructed images of two datasets measured after performing the origin scan prior to ptychography. These measurements were conducted on a 200 nm-thick Ta test pattern at 2.5 keV with the same illumination positions. The measurement and reconstruction conditions are the same as those described in the main article. To align the probe functions, their intensity centres were aligned and the corresponding sample images were shifted by the same pixel amount. Subsequently, image registration was performed for the phase images of the test chart. The two images showed offsets of 28 nm vertically and 31 nm horizontally. The correction accuracy depends on several factors, including the edge size of the reference structure, the beam size, the scan step relative to the beam size, the fitting accuracy and the position resolution of the piezo stage. In this paper, the correction accuracy was sufficient for the analysis.

S2. Reconstructed probe function

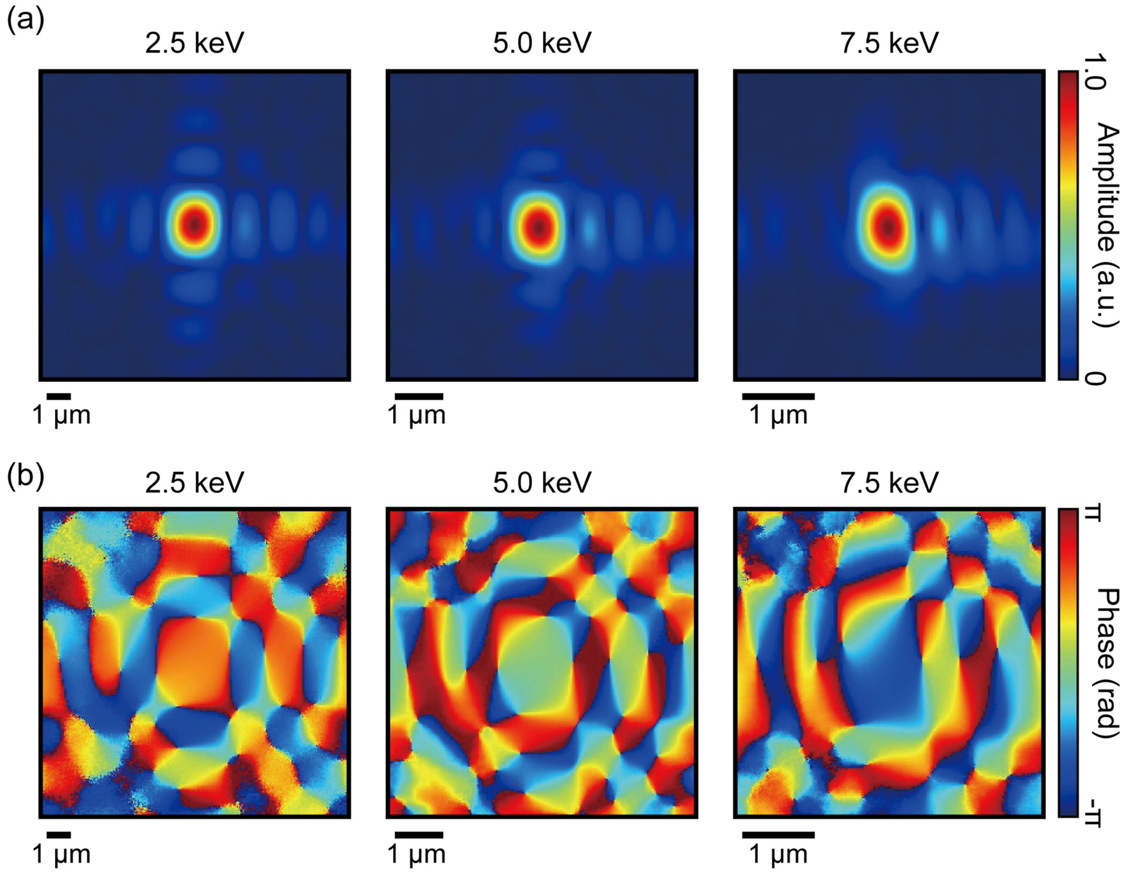

Figure S2 (a) Amplitude and (b) phase images of the reconstructed probe function (1st mode).

S3. Phase retrieval transfer function

The spatial resolution of the reconstructed phase image was assessed by utilizing the phase retrieval transfer function (PRTF). PRTF is defined as follows:

$$PRTF(\boldsymbol{q}) = \frac{\left\langle \sqrt{\sum_m \left|\mathcal{F}\left[O^{reconst}(\boldsymbol{r}-\boldsymbol{r}_j)\, P_m^{reconst}(\boldsymbol{r})\right]\right|^2} \right\rangle_{\boldsymbol{q}_{\boldsymbol{\theta}},j}}{\left\langle \sqrt{I_j^{meas}(\boldsymbol{q})} \right\rangle_{\boldsymbol{q}_{\boldsymbol{\theta}},j}}$$

where $\boldsymbol{q}$ represents the spatial frequency, $\boldsymbol{r}_j$ represents the scan centre position at the *j*-th scan, $O^{reconst}(\boldsymbol{r}-\boldsymbol{r}_j)$ represents the reconstructed object, $P_m^{reconst}(\boldsymbol{r})$ represents the reconstructed probe at the *m*-th mix-state mode, $\mathcal{F}[]$represents the Fourier transform operator , I_j^{meas} represents the measured diffraction pattern at the *j*-th scan, and $\langle\cdot\rangle_{\boldsymbol{q}\boldsymbol{\theta}}$ and $\langle\cdot\rangle_j$ denote averaging over the angular direction of spatial frequency and over the scanning points, respectively. The full-period spatial resolution is defined as the inverse of the spatial frequency at which the PRTF curve drops below the 1/e criterion. The PRTF curves of the 200 nm-thick Ta test chart at each X-ray energy are shown in Fig. S3(a), whereas the energy dependence of the PRTF curves for the $CaSO_4 \cdot 2H_2O$ particles is shown in Fig. S3(b). These findings not only validate the spatial resolution analysis but also underscore the system's performance across different X-ray energy levels.

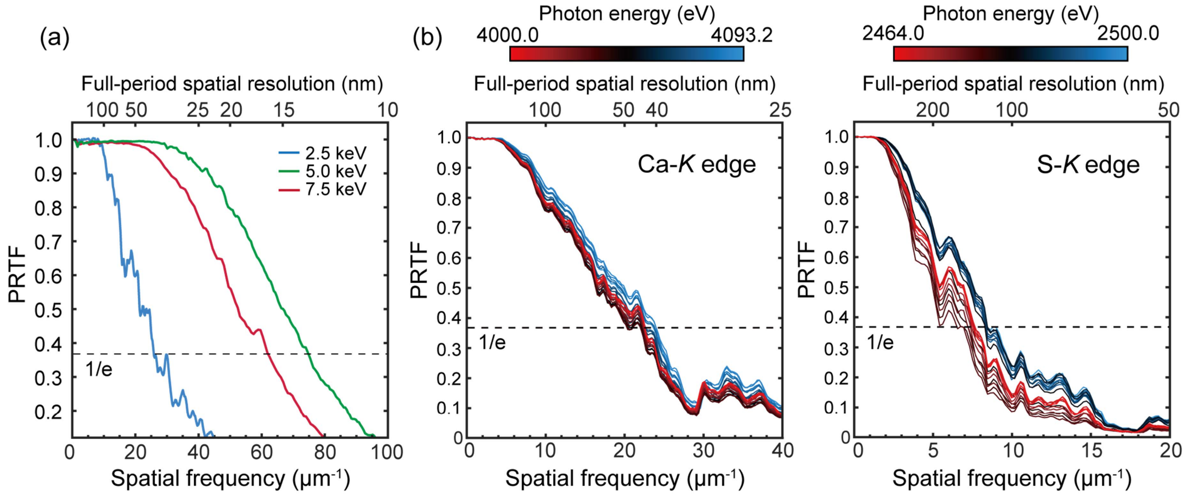

Figure S3 (a) PRTF curves for Ta 200 nm-thick test chart at 2.5, 5.0, and 7.5 keV. (b) Energy dependence of the PRTF curves for $CaSO_4 \cdot 2H_2O$ particles: (left) near the Ca *K* edge, (right) near the S *K* edge. The black dotted line represents the 1/e threshold.

S4. Line profile of the 200 nm-thick Ta test chart

The spatial resolution was evaluated from the line profiles of edge structures. Fig. S4(a) shows the phase image of the 200 nm-thick Ta test chart and Fig. S4(b) presents its edge profile fitted with an error function. The spatial resolutions estimated from the FWHM were 34.3 nm at 2.5 keV, 13.6 nm at 5.0 keV and 15.4 nm at 7.5 keV.

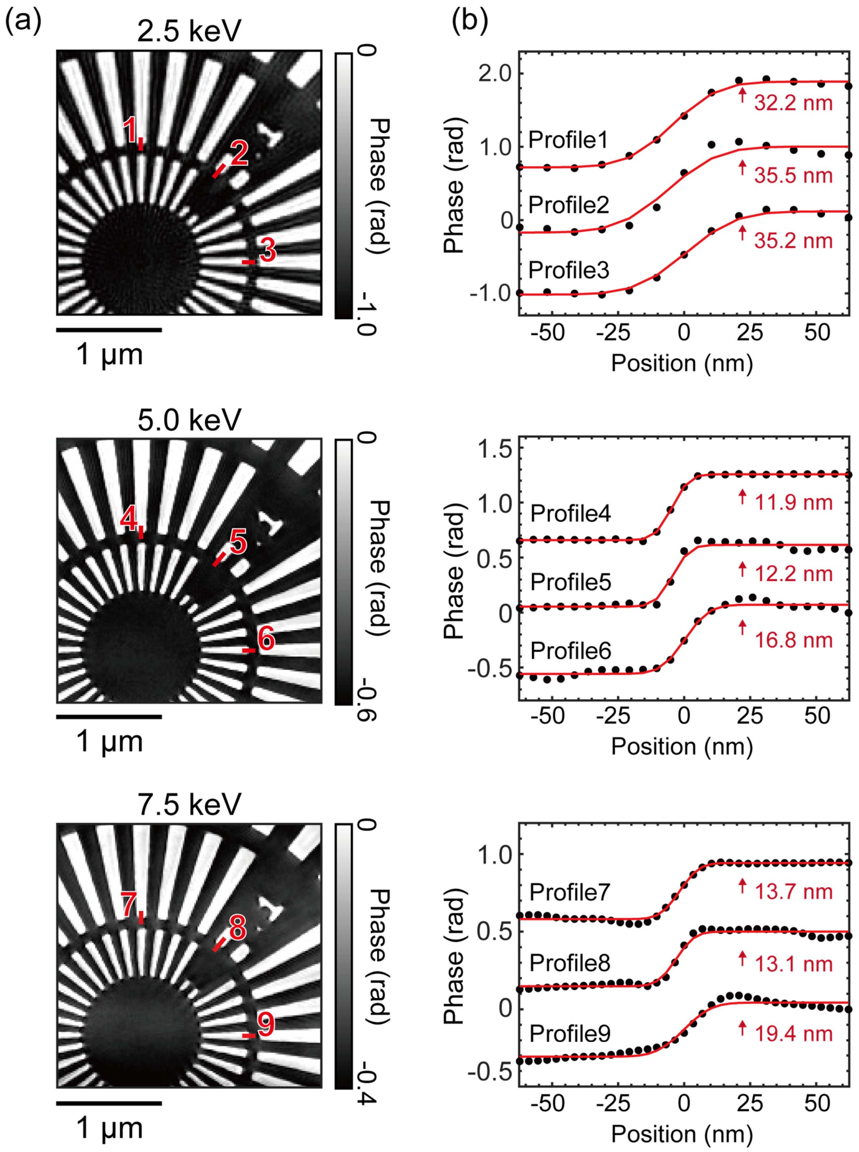

Figure S4 Reconstructed (a) phase images of the 200 nm-thick Ta test chart and (b) cross-sectional profiles of the edge structure along red lines in the reconstructed images. The FWHM values, obtained by fitting with the error function, are also displayed.

S5. $CaSO_4·2H_2O$ particles

The $CaSO_4·2H_2O$ (gypsum) used in this study can dehydrate under vacuum or low-humidity conditions to form $CaSO_4$ (anhydrite) or $CaSO_4·1/2H_2O$ (bassanite), and can rehydrate depending on the water vapour pressure (Carbone *et al.*, 2008; Tang *et al.*, 2019). The sample used in this study had been stored for several months under both vacuum (~1 Pa) and ambient conditions prior to its introduction into the sample chamber. Therefore, dehydration and rehydration may have occurred during this period. Figs. S5(a) and S5(b) show SEM images of the sample immediately after preparation and after the ptychography measurement, respectively. Crack-like fine structures were visible in Fig. S5(b), and these cracks were already present during the initial ptychography measurement. Based on previous reports of crack formation in $CaSO_4·2H_2O$ during dehydration (Brantut *et al.*, 2012), the cracks in our sample were likely caused by dehydration. From the above discussion, the sample may not be pure $CaSO_4·2H_2O$ but rather belong to the $CaSO_4$ family. For consistency, however, it is referred to as $CaSO_4·2H_2O$ throughout this paper.

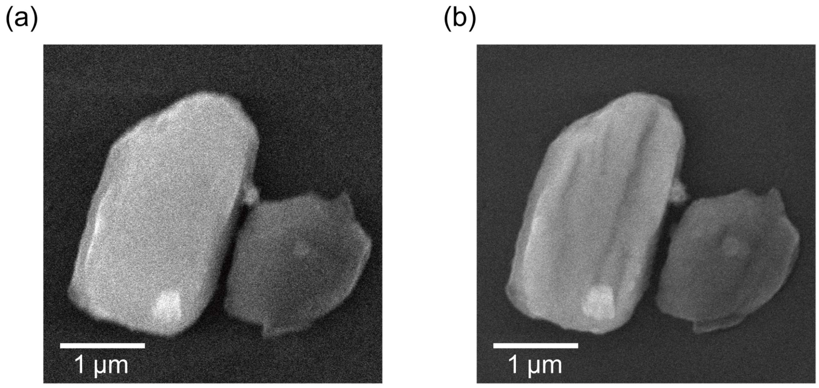

Figure S5 SEM images of the $CaSO_4·2H_2O$ particles: (a) immediately after deposition onto the SiN membrane and (b) after the ptychography measurement.
